# LCZ696, an angiotensin receptor neprilysin inhibitor (ARNI): clinical development in heart failure and hypertension

**DOI:** 10.1186/1471-2210-11-S1-O3

**Published:** 2011-08-01

**Authors:** Martin P Lefkowitz

**Affiliations:** 1 Cardiovascular Metabolism, Novartis Pharmaceuticals Corporation, East Hanover, NJ 10901, USA

## Background

Natriuretic peptides have potent natriuretic and vasodilator properties, reduce sympathetic drive, inhibit aldosterone secretion and have antiproliferative and antihypertrophic properties [1]. Increasing the concentration of natriuretic peptides through neprilysin (NEP) inhibition thus represents a therapeutic approach with the potential to confer cardiac, vascular and renal protection. Prior research, however, has suggested that the clinical benefits from NEP inhibition can be best leveraged if the renin-angiotensin-aldosterone system (RAAS) is simultaneously inhibited.

## Results

LCZ696 is a first-in-class angiotensin receptor neprilysin inhibitor (ARNI) which provides concomitant inhibition of NEP and the angiotensin receptor. Ingestion of LCZ696 delivers systemic exposure to AHU377 (which is then rapidly metabolized to LBQ657, a specific NEP inhibitor) and to valsartan (an angiotensin II receptor blocker). LCZ696 treatment is associated with dose-dependent increases in plasma cGMP, renin activity and angiotensin II, consistent with the drug’s dual mechanism of action; a dose of 200-400 mg LCZ696 achieves approximately 90% of its maximal NEP inhibition [2].

In a large Phase 2 study in hypertensive patients (n=1328), LCZ696 400mg and 200mg once daily reduced mean office systolic and diastolic blood pressure (BP) by an additional 6/3 and 5/3 mmHg compared with valsartan 320 and 160 mg, respectively (both *P*<0.05); 24-hour ambulatory BP monitoring results were consistent with office BPs. Pulse pressure was also lowered to a greater extent with LCZ696 than the respective valsartan groups (both *P*<0.05) [3]. In chronic heart failure patients (n=27), after 3 weeks treatment, LCZ696 200 mg bid increased plasma cGMP levels while decreasing plasma NT-proBNP and plasma aldosterone levels (all *P*<0.01) as compared to baseline values (4). The Phase III PARADIGM-HF study is evaluating whether LCZ696 is superior to enalapril in delaying the time to cardiovascular mortality or first occurrence of heart failure (HF) hospitalization in patients with HF and reduced ejection fraction [4]. The Phase II PARAMOUNT study is evaluating LCZ696 for HF patients with preserved ejection fraction [5]. Both studies are ongoing.

## Conclusion

Dual inhibition of neprilysin and the angiotensin receptor with LCZ696 may represent an attractive therapeutic approach for a range of cardiovascular diseases, including hypertension and heart failure, in which vasoconstriction, volume overload and neurohormonal activation play a part in pathophysiology.
